# Overview of the Current Literature on the Most Common Neurological Diseases in Dogs with a Particular Focus on Rehabilitation

**DOI:** 10.3390/vetsci9080429

**Published:** 2022-08-13

**Authors:** Giuseppe Spinella, Piera Bettella, Barbara Riccio, Samuel Okonji

**Affiliations:** 1 Department of Veterinary Medical Sciences, University of Bologna, Ozzano dell’Emilia, 40064 Bologna, Italy; 2 Department of Veterinary Sciences, University of Turin, Largo Paolo Braccini 2, 10095 Grugliasco, Italy

**Keywords:** rehabilitation, intervertebral disc herniation, degenerative myelopathy, fibro-cartilaginous embolism, polyradicoloneuritis, physiotherapy, dog

## Abstract

**Simple Summary:**

This paper aims to report an overview of the most common neurological diseases (intervertebral disc herniation, degenerative myelopathy, fibrocartilaginous embolism, and polyradiculoneuritis), with a main focus on rehabilitative options and outcomes, reported in recent veterinary literature. Literature seems to be positively oriented on the efficacy of the rehabilitation approach, reporting a careful and prudent choice of the protocol to be applied for the correct recovery of the patient. However, blinded, controlled, prospective studies are still necessary, above all for degenerative myelopathy, fibrocartilaginous embolism, and polyradiculoneuritis.

**Abstract:**

Intervertebral disc herniation, degenerative myelopathy, fibrocartilaginous embolism and polyradiculoneuritis often affect dogs; and physiotherapy may improve the patient’s quality of life and/or reduce recovery times. The aim of this review was to evaluate the current scientific outcomes on these four neurological diseases and on their physiotherapy approaches. From the analysis of the published articles, it emerged that intervertebral disc herniation can be treated, with different rates of success, through a conservative or a surgical approach followed by physiotherapy. The literature is generally oriented toward the efficacy of the rehabilitation approach in this specific canine disease, often proposing intensive post-surgery physiotherapy for the most severe conditions with the absence of deep pain perception. When degenerative myelopathy, fibrocartilaginous embolism or polyradiculoneuritis occur, the existing literature supports the use of a physiotherapeutic approach: allowing a delay in the onset and worsening of the clinical signs in degenerative myelopathy, physical improvement, and, sometimes, complete remission during fibrocartilaginous embolism or acute idiopathic polyradiculoneuritis. However, papers on rehabilitation in dogs affected by polyradiculoneuritis are currently limited to single clinical cases and further blinded, controlled, prospective studies are still advisable for all four neurological diseases.

## 1. Introduction

Physiotherapy is a branch of veterinary medicine that, through a multimodal approach (manual therapies, exercises, and modalities) aims to restore, maintain or improve optimal motor function in patients with orthopedic and neurological damage, in geriatric patients or dogs in a weight control program; prevent or delay the onset and progression of clinical signs associated with specific degenerative diseases; prevent complications due to excessive immobility or disuse of limbs; and optimize athletic performance and prevent injuries in sports and work animals [1].

The oldest references on the application of physiotherapy in the human field date back to 460 before Christ, when Hippocrates, and later Galen, proposed treatments, such as massage and hydrotherapy, for their patients [2]. Although physiotherapy plays a fundamental role in the recovery of dogs with orthopedic disorders, patients with neurological problems particularly benefit from rehabilitation therapy. Indeed, nervous system dysfunctions can lead to primary and secondary complications, which in specific cases could make recovery extremely challenging. The neurological patient is often forced to recumbency with the risk of developing sores or aspiration pneumonia, so planning a personalized and timely rehabilitation protocol is necessary for proper daily management and recovery.

The purpose of this paper is to report an updated review on the most common neurological pathologies of dogs: intervertebral disc herniation, degenerative myelopathy, fibro-cartilaginous embolism, and polyradiculoneuritis, with a particular focus on rehabilitation. 

## 2. Materials and Methods

The selection of papers was performed in December 2021 using the following databases: PUBMED, Elsevier Science Direct, and Google Scholar. Published papers were identified, selected, and analyzed by different combinations of specific keywords: rehabilitation, dog, canine, neurologic disease, intervertebral disk disease, intervertebral disk herniation, intervertebral disk extrusion, fibrocartilaginous embolism, degenerative myelopathy, and/or polyradiculoneuritis. Specialized books on veterinary rehabilitation were also consulted. 

Ninety papers were selected, but only 56 were analyzed for this review (after excluding papers not specifically related to the issues of this review); moreover, 6 books were consulted.

## 3. Acute Spinal Injuries and Aims of Rehabilitation

Acute spinal injuries are very common in veterinary medicine. The most common causes in dogs include intervertebral disc herniation, traumatic injuries (vertebral fractures or luxations, concussions, compressions or lacerations of the spinal cord), and fibrocartilaginous embolism (FCE), which can lead to an ischemic phenomenon [3].

Acute spinal damage can generate primary or secondary effects. The primary effect is the direct result of trauma caused to tissue resulting in neuronal destruction, hemorrhage, and ischemia. The secondary effect is a consequence of the primary effect but is characterized by a series of cascading events related to the destruction of the microvascular bed, cytotoxicity, liberation of free radicals, inflammation, and necrosis [3].

When a spinal cord injury occurs, the objectives of physiotherapy are as follows: (a) reduction of edema, inflammation, and pain, (b) prevention or reduction of muscle atrophy and muscle contractures due to denervation or disuse, and (c) recovery of nerve function via facilitation of neuroplasticity.

The central nervous system has the ability to adapt and alter its structure and function in response to a variety of external and internal stimuli. Neural plasticity is the mechanism by which the central nervous system (CNS) encodes experiences and learns new behaviors. Through this mechanism, during rehabilitation, the damaged system relearns the lost behavior. Indeed, the predominant use of a specific part of the body means that its representation in the motor cortex and somato-sensory cortex expands in the surrounding areas [4,5,6]. Specific neurotrophins, such as cerebral neurotrophic factor (BDNF) and neurotrophin 3 (NT-3), are endogenous promoters of neuroplasticity that seem to stimulate cell survival and facilitate axonal growth. These neurotrophins are inhibited in spinal lesions [7]. The fastest and most effective method of increasing BDNF levels is through exercise, especially if this is started shortly after an injury, to stimulate direct neuroplasticity when the nervous system is ready [8]. 

It is precisely “activity-dependent plasticity”, the mechanism on which physiotherapy intervention focuses through the choice of exercises, which will stimulate the CNS in a targeted way, so as to normalize the motor and sensory function [9,10,11]. Physical exercise, whether actively or passively performed, has a great impact on cellular and molecular functionality, promoting “activity-induced plasticity” [9,11].

### 3.1. Intervertebral Disc Herniation: Classification

Intervertebral disc herniation is a disease that affects the intervertebral discs and leads to extrusion of the nucleus pulposus (Hansen disc herniation type 1) or protrusion of the annulus fibrous (Hansen disc hernia type 2), invading the vertebral canal and resulting in the compression of the medulla. Clinical signs include pain, kyphosis, proprioceptive ataxia, paraparesis, paraplegia with or without loss of deep nociception, and other neurological damage.

The degeneration of intervertebral discs occurs with age and is considered a physiological process in aging dogs. In 1952, Hansen described two types of discal degeneration, chondroid degeneration (chondroid metaplasia) or Hansen type 1 and fibroid degeneration (fibroid metaplasia) or Hansen type 2 [12].

The term discal extrusion refers to the leakage of material from the nucleus pulposus through a damaged annulus fibrosus (normally in its dorsal portion where this is thinner). Disc extrusion is usually associated with chondroid metaplasia (Hansen type 1) whereby the forces generated by the degenerated nucleus pulposus cause the rupture of the annulus fibrosus. Extruded material can result in mild to severe compression, sometimes leading to spinal cord contusion. In 1970, Griffiths described for the first time a type of disc extrusion not associated with degeneration, which occurs as a result of trauma or intense exercise, and which involves medullary contusions with limited or no medullary compression. The term for this condition is “acute non-compressive nucleus polposus extrusion”. Since there is generally no material to be removed in the vertebral canal, normally this type of lesion does not require surgery; the damage is caused by the greater extrusion force, not by medullary compression; therefore, the most indicated therapy is the conservative approach with a specific physiotherapy and rehabilitation program [13]. In severe cases, myelomalacia can develop, and this condition could become fatal if nerves involved in breathing are affected because of respiratory arrest [14].

Fibroid degeneration is instead an aging process that occurs in middle-aged to old dogs (seven years and older) and mainly non-chondrostrophic breeds. This process involves an increase in the fibrous tissue of the pulposus nucleus resulting in degeneration of the fibrous anulus, which can occur in any tract of the spine. This type of degeneration leads to a protrusion in the dorsal direction of the fibrosus nucleus, weakening of the fibrous anulus, and dorsal protrusion of the intervertebral disc. In total, 40–60% of dogs older than seven years tend to show biochemical signs of nucleus pulposus degeneration, such as loss of glycosaminoglycans, and 10–30% show macroscopic signs of disc protrusion [15]. Discal protrusion mainly affects non-chondrostrophic breeds (e.g., German shepherds, dobermanns) and is associated with fibroid metaplasia (Hansen type 2). In dogs with disc protrusion, the symptoms occur more slowly and gradually [16].

#### 3.1.1. Cervical Disc Herniation: Conservative or Surgical Treatment and Rehabilitation

Cervical disc herniation represents about 25% of the clinical cases of disc herniation and may present with cervical pain and muscle fasciculations without neurological deficits. The lower incidence of neurological deficits, when compared to patients with thoracolumbar disc herniation, seems to be due to the size of the vertebral canal, wider in its cervical portion, allowing a greater amount of disc material to herniate without significantly compromising the spinal cord. Although infrequent, cases of ataxia with tetraparesis or even tetraplegia may also occur. 

Both type 1 and type 2 Hansen hernias can occur at the cervical level, but type 1 hernias are certainly more frequent in both small and large sized dogs, especially in the final part of the cervical tract (C5/C7) [15].

In patients at the first episode of spinal pain or with mild neurological deficits, a conservative approach is generally realized. The use of analgesics, without confining the patient, can be counterproductive as it could lead the dog to increase the degree of activity with possible worsening of the disc extrusion. 

Levine and colleagues performed a retrospective study in 2007 to assess the effectiveness of conservative therapy in dogs with disc herniation. After selecting 87 dogs with suspected cervical hernia, a survey was administered to the owners, with questions about the effects of the therapy, with particular attention to possible relapses and quality of life. Dogs in the study were treated with NSAIDs, corticosteroids, and cage rest, and the type of physiotherapy was not specifically investigated. In total, 54.7% of the dogs had complete recovery, 30.9% showed a recurrence of clinical signs, while in 14.4% of patients, therapy had no positive effect. The study found that the administration of NSAIDs appeared to be associated with greater success and a better quality of life, while corticosteroids and the duration of cage rest did not seem to significantly affect the prognosis [17].

The surgical approach is recommended in patients with severe neurological deficits and cervical pain or recurrent episodes of neck pain [16].

In the case of dogs who have paraplegia without deep pain perception, the success rate of surgery is generally reported as lower than 60%, with persistence of incoordination and paresis [18]. This aspect underlines the need for a well-defined physiotherapy program to overcome these difficulties [19].

In 2019, a retrospective study performed by Jeong and collaborators considered 58 dogs with cervical disc herniation submitted to surgery for spinal cord decompression with a ventral slot. The dogs were divided into two groups: one group was post-operatively treated with a physiotherapy protocol (group R) (34 dogs), and there was a control group of 24 dogs that did not receive a post-operative physiotherapy approach (group NR). Since functional recovery depends on the initial clinical conditions, each group was further subdivided into five subgroups based on the clinical findings reported during the neurological examination prior to surgery [19]. Before starting physiotherapy, all dogs in the R group were evaluated for muscle tone, superficial and deep pain perception, and the ability to maintain an autonomous standing position. The rehabilitation protocol included electrostimulation with a portable TENS (twice a day for 30 min), infrared therapy (twice a day for 15 min), and passive exercises to support a standing position with slings and a physioball [19]. When the patient was able to walk, an underwater treadmill (UWTM) was also introduced, gradually increasing the speed and reducing the water level. The results showed that 79.41% of patients in the R group had excellent neurological recovery, and 11.76% had poor recovery; while in the NR group, good neurological recovery was observed in 62.5% of cases, which was significantly lower (*p* < 0.05) than in the R group; while 16.67% reported no improvement. The combination of surgery with a physiotherapy protocol could consequently improve the recovery rate of neurological function, and the success of therapy was highly influenced by the patient’s pre-clinical condition [19].

#### 3.1.2. Rehabilitation of Thoracolumbar Disc Herniation

Dogs with a thoracolumbar disc herniation may show a variety of symptoms, such as pain and neurological deficits ranging from mild proprioceptive ataxia to paraplegia, with or without deep pain perception. Dogs with disc herniation generally show signs of upper motor neuron disease, but lower motor neuron injury signs are also possible due to spinal shock.

The most affected breeds are dachshunds, beagle, jack russell, and pekingese. Some clinicians suggest limiting physical activity in chondrostrophic breeds, in order to prevent the onset of a disc herniation. However, in 2016, a study performed by Packer and collaborators assessed the prevalence of disc herniation in six varieties of dachshunds with the aim of potentially identifying the risk factors associated with the development of the disease. It was found that the dogs that performed at least one hour of daily physical activity were less prone to herniation than dogs that performed less than 30 min of activity per day and were not allowed to jump [20].

Long-term complications associated with spinal cord injuries caused by disc herniation include urinary and faecal incontinence, progressive neurological deficits, and self-mutilation [21].

Conservative therapy is used in animals that have only minor neurological deficits, spinal hyperesthesia, or that cannot be anesthetized. Cage rest is of fundamental importance both to prevent other disc material extrusion and to prevent self-trauma due to poor coordination [15].

Regarding surgical treatment, it has been assumed that early surgical decompression within 48 h leads to a higher probability of success [22], although some papers have shown that there is no real scientific evidence about this assumption [23]. Several surgical options can be considered to perform a thoraco-lumbar decompression, i.e., hemilaminectomy, mini-hemilaminectomy, and dorsal laminectomy. Hemilaminectomy is the most frequently performed, as it allows direct access to the ventral and lateral portions of the vertebral canal with the possibility of herniated material ablation for complete spinal cord decompression. It also allows access to the intervertebral disc to make the fenestration, in order to prevent further material invading the vertebral canal in the post-operative period. The rate of recurrence is about 17.8%, and this rate can be reduced to 7.4% if the surgeon performed multiple preventive fenestrations [24].

In the veterinary literature, the scientific evidence that can guide the clinician towards a surgical rather than conservative approach in the treatment of the intervertebral disc herniation is rather limited since most clinical trials are retrospective and there are no randomized double-blind trials comparing the outcome of two different approaches [23].

Although gait recovery is achieved with both a conservative and surgical approach, the literature shows a faster and more complete recovery after decompressive surgical treatment (15 vs. 84 days, post-surgery and conservative, respectively) [25]. However, neurosurgeons generally recommend surgical decompression in dogs with non-ambulatory paraparesis due to a compressive thoracolumbar disc herniation [23]. This belief is supported by a meta-analysis carried out by Langerhuus and Miles, who analyzed the results of all studies published between 1983 and 2012 concerning dogs with disc herniation treated surgically or conservatively. Their research showed that 93% of dogs with non-deambulatory paraparesis or paraplegia with intact deep pain perception regained the ability to walk after decompressive surgery, while dogs treated conservatively achieved the same result only in 79% non-deambulatory paraparesis and 62% paraplegia with intact deep pain perception [25]. However, the number of dogs treated conservatively was considerably lower than the number of dogs treated surgically (113 vs. 1500); therefore, the results could be affected by this imbalance, in favor of surgical approach [23]. 

The surgical approach is very effective in relieving pain and correcting neurological deficits, but some patients may suffer from muscle weakness and incoordination, which can be quickly corrected with a timely physiotherapy approach.

In rehabilitation, a combination of therapeutic interventions appears to have a synergistic effect on functional recovery; a multimodal approach includes manual therapy, passive or active exercises, depending on the degree of motor function and the sensitivity of the patient, modalities (neuromuscular electrical stimulation, laser, ultrasound, etc.), and hydrotherapy [26].

The most indicated therapeutic exercises and protocols for rehabilitation in dogs with disc herniation vary from patient to patient and the site of the injured spinal tract. In dogs that submitted to thoracolumbar decompressive surgery, priority will be given to exercises aiming to improve proprioception, coordination, and muscle strength [16].

As generally reported in previous reviews and specialized books, the rehabilitation protocols for patients previously submitted to surgical treatment may include different exercises depending on the ability and motivation of the patient. If a surgical approach has been performed, cryotherapy is recommended in the post-operative period close to the thoracolumbar incision site (20 min twice or three times per day), immediately after surgery; while 24 h after surgery, it is possible to start with specific exercises, such as maintaining the assisted standing position on non-slip surfaces and passive range of motion (PROM) exercises. Physiotherapy can be accompanied by daily 20-min sessions of Transcutaneous Electrical Nerve Stimulation (TENS) and/or laser therapy, NMES (Neuromuscular Electrical Stimulation), and massages. For pain control, NSAIDs or opioids may be administered. After about one week and with the improvement of the physical condition, the exercises with the support by the therapist with slings or bands will be gradually reduced, and weight support can be obtained with the use of a physioball. Once the complete healing of the surgical incision is realized, hydrotherapy is introduced (for example, with 3–4 min of swimming or short walks on the UWTM), gradually increasing the duration of these activities. Exercise frequency is increased after 2–4 weeks from the surgery, when support by the operator can be considered minimal: short walks and exercises to improve coordination and balance are gradually introduced (5–10 repetitions once or twice per day). The UWTM can be alternated with the dry treadmill and at the end of each physiotherapy session, the patient should receive a 20-min session of cryotherapy to promote relaxation. At 4–6 weeks from surgery, if the patient is able to maintain his/her posture, it will be possible to increase the difficulty of the exercises: keeping the station on proprioceptive tablets, climbing or descending steps, and swimming sessions lasting 25–30 min. Rest sessions to avoid excessive fatigue of the patient are always mandatory. Throughout the rehabilitation, sessions of cryotherapy will continue after the exercises, NMES to epaxial muscles and, when necessary, anti-inflammatory drugs and/ or analgesics [16,27].

A 2018 study determined whether obesity could affect the time needed for functional recovery in dachshund dogs, with maintained deep pain perception and treated with physiotherapy after hemilaminectomy for intervertebral disc herniation. The percentages of lean and fat mass were measured by densitometry at the beginning of the study and after 12 weeks. An intensive physiotherapy protocol was performed twice per day in weeks 1, 2, 4, and 6 after surgery with progressive difficulty. All dogs included in the study were able to maintain a standing position in about 20 days, without any association between the body condition score or percentage of fat mass and post-operative outcome; instead, patients with a higher disability rate in the pre- and post-operative period, regardless of BCS, showed a longer functional recovery (>30 days) [28].

Several studies have also investigated the positive effect (anti-inflammatory, analgesic, and trophic) of photobiomodulation in dogs. In 2017, a prospective study performed by Bennaim and collaborators compared three groups of non-ambulatory dogs after surgery of hemilaminectomy for disc herniation: the first group of dogs was treated only with a “Sham” laser (without emission, placebo group), the second group with physiotherapy and the laser Sham treatment, and the third with standard post-operative physiotherapy and laser therapy. Bennaim and colleagues found no difference in the time needed for the recovery of the autonomous standing position and walking in the three groups within the limited evaluation post-operative time of the first 10 days [29]. This is in contrast to a previous retrospective study, which supported the effectiveness of laser therapy as a means to accelerate healing [30].

A more recent retrospective study performed by Bruno et al. compared the time needed for gait recovery following surgery for intervertebral disc extrusion in two groups of dogs: the first group was submitted to physiotherapy in combination with laser therapy, and the second one only to physiotherapy [31]. The physiotherapy protocol began within five days after surgery in both groups, and dogs were included if they received at least 14 days of physiotherapy. Two sessions per day on five days per week were performed on each dog, in which they received massages, PROM, and other passive and active exercises such as balance, cavaletti rails, and sit to stand. Seven days after surgery, patients also started UWTM sessions with the water level at the height of the greater trochanter of the femur. 

Dogs in group 1 also received laser therapy sessions with a point-to-point technique, applied transcutaneously on clipped hair and with a 45°-degree latero-medial probe inclination. The results showed that dogs with deep pain perception, treated with laser therapy associated with the physiotherapy protocol had a lower improvement in the neurological status than the control group, but this difference was not statistically significant; however, all dogs in the laser group regained autonomous ambulation, unlike the group with only physiotherapy [31].

Regarding the use of treadmills for ambulation recovery in neurological patients, a prospective clinical study performed in 2021 by Martins and colleagues compared the activity on a traditional treadmill to a treadmill system with body weight support. The study involved tetraplegic or paraplegic dogs with no superficial pain perception but with deep pain perception. All dogs were previously treated surgically. The dogs were divided into two groups of 10 patients each. Patients from both groups underwent two weeks of exercises on the UWTM, interference electrical stimulation (IES), and functional electrical stimulation (FES). Four weeks after the beginning of the rehabilitation, 60% (6/10) of the dogs from the group that used the treadmill with weight support recovered their motor functions, whilst the same result was observed in only 10% of the dogs (1/10) in the group that walked on the traditional treadmill. After two weeks of different protocols on the treadmill, all dogs in the two groups followed a unique treadmill protocol with weight support. At the end of the study, the results showed that ambulation was achieved in 100% of the dogs included in the “body weight-supported” treadmill training group (in a 4.6-week period), whilst in the group of patients that started rehabilitation with the traditional treadmill, only 78% of the dogs had functional recovery (in a 6.1-week period) [5].

Another study that supported the importance of rehabilitation after surgery for intervertebral disc herniation was performed in 2015 by Hodgson and colleagues on 248 dogs weighing less than 20 kg [32]. Patients were divided into two groups according to whether or not there was a specific post-hemilaminectomy physiotherapy protocol. The study considered the elapsed time for the reappearance of deep pain perception and to obtain the ambulatory status. After 24 h, the first neurological assessment was performed. Sessions of cryotherapy, laser therapy, and PROM were performed on dogs in both groups until discharge. Laser therapy was performed once per day. Dogs in the rehabilitation group performed exercises in a specialized clinic and at home with the owner. Patients were taken to the clinic to follow the therapies two or three times per week for 4–6 weeks. 

At the end of the study, 33% of the animals in the group with physiotherapy completely recovered (grade 5 of the modified Frankel scale), while 53% reached grade 4 (ambulation with ataxia); in the control group, only 9% of dogs had a complete recovery (grade 5 of the modified Frankel scale), and 82% reached grade 4. The control group took less time to start walking again and regain conscious proprioception; however, in the rehabilitation group, there was a greater number of dogs with a loss of deep pain perception, and this may have adversely affected the calculated average recovery time. Since this was a retrospective study, dogs were not randomly included in the two groups, it is therefore likely that the dogs in the rehabilitation group were those in more severe pre-operative conditions and, as a result, more prone to start physiotherapy. The study therefore shows that rehabilitation may not accelerate recovery time, but based on the number of dogs that reached grade 5 of the modified Frankel scale, it is more likely to contribute to a complete recovery of functionality [32].

Patients with no deep pain perception can particularly benefit from physiotherapy. Loss of nociceptive perception is generally associated with a poor prognosis for motor function recovery, as this condition is a highly likely sign of complete spinal cord injury. If the spinal injury is complete, the purpose of the physiotherapy protocol is mainly aimed at acquiring the so-called “spinal gait” or “spinal walking”.

A retrospective study performed by Gallucci et al. on 81 dogs with complete spinal injuries and a lack of deep pain perception showed that the application of an intensive physiotherapy protocol could lead to the development of an involuntary unassisted gait in 59% of the patients. In total, 66% of the dogs considered in the study had a thoraco-lumbar disc herniation. At the end of the physiotherapy course, the dogs were divided into two groups based on the obtained result (presence or absence of spinal walking). The study results showed that young age and an early start of physiotherapy were associated with a better result [33].

A more recent retrospective study performed by Martins and collaborators also analyzed the data of 367 paraplegic dogs suffering from Hansen type 1 thoracolumbar disc herniation, in order to evaluate whether an “intensive” rehabilitation protocol can correctly stimulate “activity-induced neuroplasticity”, improving walking ability more rapidly than with a standard physiotherapy protocol or spontaneous healing [6]. For this study, only dogs submitted to hemilaminectomy surgery within 3–5 days from the onset of symptoms were selected and classified before and after surgery, using a modified Frankel scale, in dogs with grade 0 (absence of deep pain perception) or with grade 1 (with deep pain perception, but with absent or reduced flexor reflex). The patients were then divided into two groups: the first, consisting of 262 dogs, was submitted to an intensive physiotherapy program (IFP), and the second, Control Group (CG), consisting of 105 dogs, was obtained by retrospective evaluation of previous neurological cases managed within the same clinic, but that had not followed an intensive protocol. The CG was generally treated with cage rest, PROM, massage, NMES, and assisted weight support. All dogs included in the IFP underwent NMES, exercises for locomotor training, functional electrical stimulation, and additional pharmacological management (4-aminopyridine for a maximum period of two months) for those patients that did not have deep pain perception after 30 days of rehabilitation. Interferential current and functional electrical stimulation was also administered to reduce pain and increase muscle strength and neural connections. Patients were neurologically evaluated every 5–7 days for the presence or absence of deep pain perception, flexion–extension reflexes, and the ability to maintain the upright position.

The recovery rate of ambulation in dogs of the IFP group with maintained nociception was 99.4% (167/168): 68% of these dogs started walking again in a period of time ranging between 7 and 14 days after starting physiotherapy. Of the 94 dogs included in the IFP group without deep pain perception, 58.5% (55/94) regained the ability to walk and 33.2% regained the perception of pain; 22 dogs developed a spinal gait in a maximum period of three months [6]. In conclusion, although it is difficult to determine the concrete contribution of an intensive physiotherapy protocol to ambulation recovery with a retrospective study, this method appears to indicate this approach for treating paraplegic patients [6].

However, evidence-based studies do not often support the hypothesis that intensive physiotherapy increases the recovery rate of function when a spinal injury occurs. In 2018, a randomized, blinded, prospective study was published with the aim to compare the functional recovery rate after decompressive surgery for thoracolumbar disc herniation in dogs treated with a basic rehabilitation protocol or an intensive physiotherapy program [18]. All 30 dogs included in this study had non-ambulatory paraparesis or paraplegia with deep pain perception. All dogs underwent decompression surgery within 24 h of admission, which was followed by pain-relieving therapy and treatment with cryotherapy at the incision site. The animals were then assigned to the two different rehabilitation groups in a random but stratified method, so that each group had an equal number of non-walking paraplegic or paraparetic dogs. The day after the surgery, the dogs included in the study began the physiotherapy protocol. All dogs underwent PROM exercises every 12 h. Dogs in the basic rehabilitation group were treated with cryotherapy, leashed assisted walks, and PROM. Dogs included in the group submitted to intensive physiotherapy also performed NMES sessions, assisted upright position exercises, the UWTM, and exercises for muscle strengthening on a balance board. After 14 days of hospitalization, the patients were discharged and the owners were instructed on how to perform walks and PROM exercises at home. At the time of discharge, all dogs in both groups, except one, were able to independently walk. The time and degree of recovery of the walking ability and coordination were compared, and the authors did not report statistical differences between the two groups. The authors therefore concluded that the intensive physiotherapy approach in the post-operative phase is safe and well tolerated, but no statistical differences were reported as an outcome of dogs receiving early intensive postoperative rehabilitation compared with a less intensive postoperative. The discrepancy with what emerged from other studies could be due to the reason that in this latter study, only dogs with maintained deep pain perception and non-ambulatory status for no longer than three days were included. It is therefore plausible that the application of an intensive protocol in the post-operative management of patients with intervertebral disc hernation may be more useful for patients with more severe neurological conditions [18].

## 4. Fibrocartilaginous Embolism (FCE)

Fibrocartilaginous embolism (FCE) is a non-compressive pathology of the spinal cord of vascular origin, characterized by ischemia secondary to obstruction of a medullary vessel by fibrocartilaginous material, histologically identical to the nucleus pulposus of the intervertebral disc [34]. It was first described in humans in 1961 by Naiman and, subsequently, in the dog by Griffith in 1973. Other species such as cat, horse, cattle, and pig seem to be less affected [35].

The dogs affected by the disease are generally adults, belonging to large or giant non-chondrodystrophic breeds; less frequently it is observed in young, small breed or chondrodystrophic dogs [16]. The higher incidence in non-chondrodystrophic breeds seems to be due to the reason that in these dogs, the nucleus pulposus remains in its physiological gelatinous status for a longer period of time compared to the chondrodystrophic breeds, which are more predisposed to chondroid metaplasia [36].

The onset is acute and often associated with physical activity such as running or playing [16]. Dogs generally come to the visit without obvious spinal pain, and often in the medical history, the owners have reported hearing the dog yelp or showing signs of pain at the onset of symptoms [36]. Any tract of the spinal cord can be affected, although the tracts most frequently affected are the spinal segments C6-T2 and L4-S3, and less frequently at the level of C1-C5 and T3-L3 [37]. Depending on the spinal cord segment involved, specific neurological deficits such as ataxia, paresis or paralysis may be observed, which may be symmetrical or asymmetrical [16].

Fibrocartilaginous embolism is a non-progressive disease, and neurological deficits will rarely progress after the first few hours unless an ascending or descending myelomalacia develops [36]. The diagnosis is made on the basis of the clinical signs, excluding other possible causes such as disc herniation, trauma, neoplasm or inflammation [16]. However, even for this pathology, the definitive diagnosis can only be made post-mortem.

### Physiotherapy Approach to FCE

The rehabilitation techniques applied to dogs suffering from FCE are similar to those used for patients with herniated discs and vary according to the location and extent of the damage, as well as based on the symptoms. A patient with FCE may have increased muscle tone and spasticity or decreased tone and flaccidity.

In general, the rehabilitation protocol includes electrostimulation (especially in the course of reduced muscle tone), passive range of motion (PROM) exercises, water therapy, and assisted walking. Similarly, for dogs affected by intervertebral disc disease, if the patient is unable to walk, further attention will be required for his/her management, including adequate beds to prevent the onset of pressure sores, frequent recumbency changes, and management of the bladder, which must be emptied every 6–8 h. PROM exercises and joint/muscle mobilizations are performed regularly to prevent the risk of contractures, generally 10–15 repetitions three to five times per day, associated with pedal exercises and massage movements. When the dog is standing, it will be necessary to pay particular attention that the patient loads the four limbs correctly. Hydrotherapy in the pool, with the help of life jackets if necessary, is used to enhance muscle activity: the therapist can passively move the patient’s limbs to stimulate motor memory; initially the swimming sessions will last 2–5 min, and then sessions will be about 10 min [16]. As the animal’s clinical conditions improve, exercises on the treadmill and assisted walks can be introduced.

Once the patient is able to autonomously maintain a standing position and regains the ability to walk without support, more complex exercises will be introduced, such as changes of direction during the walk, rails, and turning exercises with weave poles. Generally, the rehabilitation protocol has a mean duration of three weeks, which can be extended for those patients with a slower recovery: it is essential to pay attention to possible signs of fatigue, in order to avoid lameness or generalized pain that would decelerate the rehabilitation process [27].

In a retrospective study carried out by Gandini and collaborators, the medical records of 75 dogs diagnosed with FCE were evaluated, of which 21 were euthanized at the request of the owners and included in “group A”, on which necropsy was carried out and confirmed the diagnosis of fibrocartilaginous embolism. The remaining group of dogs in “group B” consisted of dogs whose diagnosis of FCE was made on the basis of clinical signs. The FCE course and recovery rate were analyzed and compared between the two groups. Recovery was defined as the return of the ability to correctly place limbs during walking or a standing position; slight incoordination or occasional loss of balance was considered acceptable. Patients began physiotherapy as soon as the diagnosis of suspected FCE was obtained, generally within 24–48 h from the onset of symptoms. The physiotherapy program included PROM with the patient in lateral recumbency or an assisted standing position. All patients with more severe conditions underwent hydrotherapy sessions, in which the operator manually positioned the dog’s limbs. If the animal lost the deep pain perception or showed signs of lower motor neuron disease, the affected limb was treated with electrostimulation. Finally, for patients with adequate motor functionality, assisted walks were carried out five times per day. In total, 73.4% of the dogs in group B showed a fast and almost complete recovery within two weeks, while in the remaining 26.6% of cases, it took from 15 to 45 days before the dogs were able to walk independently; one dog showed no signs of improvement and was euthanized at the request of the owner. The results of this study showed that the localization of the lesion and the involvement of the intumescences did not significantly influence the time of recovery, while the symptom most linked to a negative prognosis was the absence of deep pain perception [35]. The authors concluded that active nursing and intensive and early physiotherapy should be applied when a suspected fibrocartilaginous embolism is clinically suspected, as rehabilitation plays a crucial role in the positive outcome of the disease [35].

## 5. Degenerative Myelopathy

Degenerative myelopathy, previously called “chronic degenerative radiculomyelopathy”, is a progressive and worsening disease, observed in adult/elderly dogs (generally ≥ five years old), which affects the hind limbs and entails upper motor neuron ataxia and paresis, up to paralysis [22]. This pathology was first described in 1973 by Averill in a German shepherd dog, in which degeneration of the spinal cord, in particular of the white matter, of unknown origin was observed [22].

The disease progresses slowly; initially, the affected dogs show proprioceptive ataxia; often in the initial stages these deficits occur asymmetrically, subsequently it progresses with paresis, and finally paralysis. In most dogs, clinical signs of the disease are consistent with upper motor neuron injury, with increased spinal reflexes, proprioceptive deficits, and increased muscle tone; less frequently, decreased spinal reflexes, as well as muscle tone, have been reported [38]. An essential feature of this disease is that it does not show spinal pain. Most patients start to not ambulate within 6–9 months from the onset of symptoms, at which point many owners opt for euthanasia. Dogs that are not euthanized will experience progressive loss of hind limb spinal reflexes, paresis of the thoracic limbs, fecal and urinary incontinence, and signs of brain stem involvement such as dysphagia [16] and, sometimes, breathing difficulties [39].

This condition was first described in the German Shepherd, but it may affect many other breeds, including the Welsh Corgi Pembroke, Siberian Husky, Boxer, and Labrador Retriever [22].

A correct diagnosis of degenerative myelopathy can be challenging, as the definitive diagnosis can only be performed post-mortem through histopathological analysis of the spinal cord. As far as localization is concerned, the pathology affects the lateral and ventral bundles of the thoraco-lumbar medullary segments (from T3 to L3) [39]. The distribution of the lesions varies according to the breed: in German shepherd dogs, lesions appear discontinuous, bilateral, and asymmetrical, while in Welsh Corgi Pembroke dogs, they appear longitudinally continuous, within more defined areas [22].

Etiopathogenesis of this pathology is not clear, and various hypothetic etiologies have been taken into consideration over the years: metabolic, nutritional, vascular, immune-mediated, and/or vitamin E deficiency, but it is currently thought that the more accredited cause may be genetic. Clemmons and colleagues, in 2006, hypothesized a genetic correlation between human multiple sclerosis and degenerative myelopathy in dogs. Indeed, multiple sclerosis leads to a human leukocyte antigen (HLA) mutation in the DRB1 region, and Clemmons et al., who analyzed the gene portion of the canine leukocyte antigen (DLA) by PCR in healthy German shepherds and in German shepherds with degenerative myelopathy, observed a mutation similar to that of human multiple sclerosis [40]. Subsequently Awano, in 2009, identified a mutation affecting a single nucleotide in a specific region of chromosome 31 containing the gene that codes for superoxide dismutase (SOD-1), which is one of the most represented proteins in the CNS with specific functions of removing free radicals. In pathological dogs, a substitution from guanine (G) to adenine (A) was observed in exon 2 in position 118 (c.118G>A), which led to a missense mutation from glutamic acid to lysine in position 40 (E40K) [41].

### Physiotherapeutic Approach to Degenerative Myelopathy

Prognosis of degenerative myelopathy is poor, and most owners opt for compassionate euthanasia of their dog; nevertheless, it is always correct to advise conservative physiotherapy to improve or decelerate the evolution of the pathology and the motor skills of the dog. 

In 2006, Kathmann and colleagues performed a retrospective study in order to evaluate the effect of physiotherapy on the survival times of dogs suffering from degenerative myelopathy considering the age of the patient at the onset of symptoms and the lesion location [42].

The study involved 22 dogs, divided into three groups according to the intensity of the physiotherapy protocol: group 1 included dogs that received an intensive physiotherapy protocol, group 2 dogs were treated with a moderate approach, and finally group 3 dogs did not receive any rehabilitation treatment.

The rehabilitation protocol included passive exercises such as assisted standing position, mobilization of the joints of both hind limbs, massages on the hind limbs and paravertebral muscles (once or twice per day), and active exercises such as walking with a leash (even on different terrains such as asphalt, sand, grass-assisted, if necessary, by a harness to support the body weight), sit–stand exercises (10 repetitions twice per day), climbing stairs, and walking uphill. Hydrotherapy was also performed (UWTM or swimming pool), gradually increasing the duration. The duration and frequency of the exercises were related to the dogs’ physical status and intensity of the rehabilitation protocol. Dogs submitted to intensive physiotherapy performed active exercises 3–5 times per day, massages three times per day, and hydrotherapy once per day; dogs with a moderate physiotherapy performed three sessions of active exercises per day and hydrotherapy once per week. At the time of euthanasia, all 22 dogs were paraplegic. The outcome of this study shows that physiotherapy is a valid treatment in delaying the progression of the disease and extending survival, as the dogs included in the intensive physiotherapy protocol group had an average survival time of 255 days versus 130 days of dogs under moderate treatment, and significantly longer than the 55-day survival of the group without physiotherapy [42].

More recently, Miller and colleagues performed a retrospective study to evaluate the efficacy of photobiomodulation (laser therapy) in association with physiotherapy for the treatment of degenerative myelopathy [43]. The authors introduced laser therapy into the rehabilitation protocol because it benefits spinal cord injury treatment, related to the suppression of pro-inflammatory cytokines, the decrease in invasion by macrophages and T lymphocytes, and the enhancement of the growth capacity and axonal function [43].

Two different photobiomodulation protocols were compared. All 20 included dogs were submitted to the same rehabilitation protocol, which included two weekly sessions at the clinic and a specific exercise program to be performed at home. Rehabilitation at the clinic included different exercises: (a) passive stretching; (b) assisted standing position; (c) assisted standing position with the front or hindlimbs resting on a physioball; (d) standing and slalom between cones; and (e) treadmill in the water. Both photobiomodulation protocols were performed in non-clipped areas with the probe in direct contact with canine coat above the vertebral column and 5–7 cm to the right and left of the vertebral column, at the level of the paraspinal muscles, from the tract between T-3 up to the lumbosacral region. Dogs were divided into two groups: six dogs followed protocol A, realized with a class 3B laser set to a wavelength of 904 nm (power of 0.5 W; energy density of 8 J/cm^2^ per point) and applied following a point-to-point grid method consisting of 20 points scattered along the area to be treated; protocol B was performed on 14 dogs with a class 4 laser with a wavelength of 980 nm (power of 6–12 W, depending on size; energy density of 12–21 J/cm^2^, average over the treated area), in which the probe was moved continuously over the treatment area at a speed of 2–4 cm/s for a total treatment duration ranging from 15 s to 25 min, depending on the size of the dog [43].

At the end of the study, in dogs treated with protocol B, the time between the onset of symptoms and non-ambulatory paresis and, subsequently, euthanasia was significantly longer than that in dogs treated with protocol A. The improvement was due, according to the authors’ opinion, to the greater efficacy of protocol B (class 4 laser), which involved a higher intensity able to positively interact with the underlying tissues, realizing a real therapeutic effect. The times observed in group A, on the other hand, were comparable to those reported by Kathmann et al., 2006 and Polizopoulou et al., 2008 [42,44].

## 6. Acute Polyradiculoneuritis

Acute polyradiculoneuritis is an acute inflammatory disease, on an immune-mediated basis, which affects the axons and myelin of the spinal roots and peripheral nerves (with greater compromise of the ventral roots), and is characterized by a rapid development of generalized weakness, up to lower motor neuron tetraparesis/tetraplegia. In severe cases, respiratory muscles may also be involved, while cranial nerves (excluding facial nerves), urination, and defecation do not appear to be affected [45]. A similar condition, called Guillain–Barrè syndrome, also occurs in humans. Acute polyradiculoneuritis is one of the most commonly encountered peripheral nervous diseases in dogs [46]. According to Pellegrino and collaborators, this pathology represents 6% of neuromuscular pathologies in dogs [47], probably underdiagnosed due to an inadequate diagnostic procedure, spontaneous recovery of the patient or early euthanasia in the case of the acute onset of tetraplegia [48].

The etiology is not yet fully known, and some patients have shown clinical symptoms 7–10 days after receiving a bite from a raccoon, so in the past, the disease was also known as “Coonhound paralysis”, because it was assumed to be an immune reaction against an antigen present in the saliva of raccoons; however, the pathology is widespread even in areas where raccoons are not present [45]. Other possible etiological causes are recent vaccinations, especially against rabies and recent respiratory and gastrointestinal infections of bacterial or viral origin [46]. In humans, the development of Guillain–Barrè syndrome has been associated with infections caused by Cytomegalovirus, Epstein–Barr virus, HIV, *Campylobacter jejuni* bacterial enteritis, and other etiological agents with epitope-like antigenic characteristics present on the myelin of peripheral nerves or ranvier nodes. When these infectious agents activate the immune system, peripheral nerves are also recognized as targets [49]. In immune-mediated diseases, the target epitopes on the myelin membrane have to be accessible to antibodies because the disease may develop, this means that the epitopes must be exposed on the outer surface of the myelin membrane and that the blood–nerve barrier is damaged [46]. An excellent target for attack by the immune system seems to be the oligosaccharide portions of glycolipids; in particular, many antibodies react against glycolipid antigens, mainly represented by gangliosides [50].

Gangliosides are present on plasma membranes throughout the body but occur in higher concentrations in nerve tissues. In humans with Guillain–Barrè syndrome, the main targets seem to be GM-1 and GT1b gangliosides, while it was determined that in dogs suffering from polyradiculoneuritis the main target is GM2 ganglioside. The presence of anti-GM2 antibodies in dogs suffering from acute polyradiculoneuritis suggests that this could have a pathophysiology similar to Guillain–Barrè syndrome, acting as an interesting animal model for human research. Furthermore, anti-GM2 antibodies can be used as biomarkers for the diagnosis of the disease [51]. The ventral roots of the spinal nerves are the most affected, especially in the lumbo–sacral region, leading to axonal degeneration, paranodal demyelination, and inflammatory infiltrate. The type of involved inflammatory cell varies in relation to the duration of clinical signs: when a hyperacute evolution occurs, neutrophilic granulocytes are prevalent, while in chronic cases, lymphocytes, plasma cells, and macrophages predominate [46].

Several studies have been performed in order to determine the possible triggering agents and, in 2011, Holt and collaborators performed a retrospective study on the serum of 88 dogs, of which 44 were affected by acute polyradiculoneuritis and 44 were used as a control group, in order to evaluate the antibody titer against *Ehrlichia canis*, *Borrelia burgdorferi*, *Toxoplasma gondii*, *Neospora caninum*, *Campylobacter jejuni* or Distemper. The study found that 55.8% of dogs with acute polyradiculoneuritis had significantly higher antibody titers against *Toxoplasma gondii* than dogs in the control group [49]. However, in 2013, a study by Rupp et al., reported that only one of the 14 included dogs showed antigens against *T. gondii* [51].

In 2018, a comparison by PCR of fecal samples from 27 dogs with suspected polyradiculoneuritis with 47 healthy dogs showed a higher prevalence of *Campylobacter spp.* infection in dogs with the disease. Furthermore, a strong correlation was noted between the consumption of raw chicken meat and the occurrence of polyradiculoneuritis, as chicken meat is the most common source of *Campylobacter jejuni* in industrialized countries [52].

Research on anti-GM2 antibodies has reported good results in a study performed by Rupp and collaborators, in which a glycoarray achieved a diagnostic sensitivity of 60% and a specificity of 97% [51]. More recently, in 2021, Halstead and collaborators conducted another study in which they investigated a larger and more geographically heterogeneous group of dogs. The search for anti-glycolipid antibodies against eleven common glycolipid targets was performed through the serological analysis of 175 dogs with acute polyradiculoneuritis, 112 dogs with other peripheral, cranial or neuromuscular neurological disorders, and 226 dogs without neurological disorders. The aim of the study was to determine which of these had greater specificity as well as sensitivity for the diagnosis of polyradiculoneuritis [53]. GM1, GM2, and GA1 glycolipids were initially identified as the main target of anti-glycolipid antibodies (AGAbs) in dogs with polyradiculoneuritis. Furthermore, analysis for antibodies to GalNAcGD1a (ganglioside sharing terminal epitope with GM2), demonstrated a higher sensitivity (65.1%) and specificity (90.2%) of AGAbs anti-GM2 compared to AGAbs anti-GalNAc-GD1a (sensitivity 61.7%, specificity 89.3%), although AGAbs anti-GalNAc-GD1a were more frequently observed in non-ambulatory dogs. The study therefore confirmed the usefulness of examining the presence of AGAbs for diagnostic purposes in dogs with suspected polyradiculoneuritis [53].

### Physiotherapic Approach to Acute Polyradiculoneuritis

An intensive physiotherapy approach is a fundamental step in the treatment of the disease, especially to maintain joint mobility, avoid muscle contractures, and decrease/delay neurogenic muscle atrophy [46].

The rehabilitation plan for dogs suffering from acute polyradiculoneuritis has to be adapted to the clinical condition and ambulatory ability of each patient [54].

In the first few days (1–5 days), it will be necessary to perform manual stimulation of the flexor reflex as well as PROM exercises and massages. It may be helpful to place the dog in a standing position, maintaining this for even a few seconds.

From 5 to 21 days, if an improvement occurs, exercises will be introduced to maintain the standing position. If the patient is able to autonomously support the head, these exercises can also be performed in water, assisting the correct positioning of the limbs during UWTM activities.

During the maintenance phase (lasting 21–60 days), assisted walking exercises and UWTM walks can be gradually introduced and increased; the aim of this phase is to improve coordination and proprioception. Muscle recovery can be achieved during hydrotherapy and, when necessary, through the use of electrical muscle stimulation.

In the advanced stage, the intensity of the therapeutic sessions and the difficulty of the proposed exercises can be increased [54].

Although acute polyradiculoneuritis is a common neurological disease often admitted to rehabilitation, only a few specific studies on rehabilitation protocols have been published.

In 2015, Griffiths published a case report relating to a clinical case of a 9-year-old Border Collie brought to the visit for sudden difficulty walking with progressive worsening, up to paralysis of the hind limbs after about 6–7 days from the onset of symptoms. The forelimbs did not show neurologic changes, and the diagnostic imaging showed no signs of spinal cord compression.

The therapeutic approach included PROM exercises, massages, and an assisted standing position, first through the use of a four-wheel dog wheelchair and, subsequently, with the use of a physioball. After four weeks from the start of rehabilitation, the protocol was intensified with the inclusion of UWTM sessions, initially lasting a few minutes and then gradually increasing. Two weekly hydrotherapy sessions and passive and active exercises were performed at the clinic, associated with daily treatments performed at home by the owners. Two months after the onset of symptoms, the dog was able to walk independently, and rehabilitation was still carried out in order to improve and enhance stability and balance; the owners were also encouraged in this phase to take the dog to walk uphill or on different surfaces such as grass or sand in order to simulate a proprioceptive path [55].

Although most dogs report a total recovery, patients with more severe signs may show some residual neurological deficit, or even show no clinical improvement or reoccurrence despite a previous complete functional recovery. If involvement of the diaphragm muscles is also observed, it will be necessary to keep the dog in intensive care with ventilatory support [47].

Another case report, in 2021, described by Kraljevic and collaborators, reported the rehabilitation protocol and clinical outcome of two mixed breed dogs, females of 6 and 10 years of age, brought to visit with signs of non-ambulatory tetraparesis with maintained deep pain perception. The dogs presented lateral recumbency, unable to assume the sternal position. Proprioception was absent in the forelimbs and reduced in the hindlimbs, with the exception of the perineal reflex.

Rehabilitation included exercises at home by the owners such as massages and PROM, which were performed 2–4 times a day, in addition to electrostimulation sessions (electro-acupuncture), hydrotherapy, and proprioceptive exercises. After two weeks both dogs showed voluntary movements of the hind limbs and were able to maintain the sternal position without support. The ability to maintain the standing position and voluntary movements of all four limbs were obtained after three and five weeks, respectively. After improvement, active exercises and walks on a UWTM were introduced, such as exercises on balance-boards and Cavalletti rails. The two dogs were able to walk autonomously in 25 and 50 days, respectively [56].

## 7. Discussion

From the analysis of aforementioned studies, included in this review, it is the authors’opinion that currently the veterinary literature still lacks of high number of controlled, blinded, prospective studies that may prove the evidence-based effectiveness of rehabilitation in neurological canine disease. Nevertheless, clinical practice shows that physiotherapy represents valid support for the recovery of the most common neurological pathologies, with some distinctions for treated pathology, in relation to the typology of the intensity of the rehabilitative protocol. Most publications are related to rehabilitation in dogs affected by intervertebral disc herniation, conservatively or surgically treated. Retrospective and prospective studies on patients suffering from intervertebral disc herniation have proved that an “intensive” physiotherapic protocol could be applied when severe neurological damage (para/tetraplegia with the absence of deep pain perception) is clinically diagnosed and if surgically treated for decompression [5,6]. Conversely, when a less severe condition is clinically observed (ambulatory para/tetraparesis, non-ambulatory para/tetraparesis, paraplegia with pain perception), a “moderate” protocol could lead to a complete recovery [18]. A lack of convergence on specific modalities has also emerged with regard to the application of photobiomodulation during rehabilitation, as some authors with retrospective studies observed positive effects on treated patients [30,31], while the prospective study of Bennaim et al., although after a limited post-operative observational period of the first 10 days, resulted in no statistical differences between groups with or without laser application [32].

For other pathologies, such as fibrocartilaginous embolism, degenerative myelopathy or polyradiculoneuritis, the main and generally unique therapeutic approach is represented by physiotherapy, although this statement is generally supported only by retrospective studies [35,42,43,44,55,56]. In degenerative myelopathy, the worsening trend of disease often leads owners to opt for compassionate euthanasia, but physiotherapy associated with laser-therapy may delay the onset of the final stages of the disease, improving the duration and quality of the patient’s life. Moreover, although fibrocartilage embolism and polyradiculoneuritis are less investigated from a scientific point of view, it is nevertheless clinically established that physiotherapy can be an effective treatment and, generally, the only therapeutic strategy for a complete recovery of ambulation.

Finally, a limitation of this review could be the selection and analysis of papers, obtained by the combination of specific keywords within specific databases.

## 8. Conclusions

In conclusion, despite the necessity of further prospective studies, rehabilitation is currently a fundamental therapeutic step in the recovery of the most common neurological diseases of dogs. However, despite the presence of standardized protocols, physiotherapy has to be adapted to each individual patient based on the initial clinical condition and ambulation ability.

## Data Availability

Not applicable.
